# Truncating mutation in *TANC2* in a Chinese boy associated with Lennox-Gastaut syndrome: a case report

**DOI:** 10.1186/s12887-021-03021-3

**Published:** 2021-12-03

**Authors:** Yang Tian, Zhen Shi, Chi Hou, Wenjuan Li, Xiuying Wang, Haixia Zhu, Xiaojing Li, Wen-Xiong Chen

**Affiliations:** Department of Neurology, Guangzhou Women and Children’s Medical Center, Guangzhou Medical University, 9# Jin Sui Road, 510623 Guangzhou, Guangdong Province PR China

**Keywords:** *TANC2*, Epilepsy, Lennox-Gastaut syndrome, Case report

## Abstract

**Background:**

Lennox-Gastaut syndrome (LGS) is a severe epileptic encephalopathy that can be caused by brain malformations or genetic mutations. Recently, genome-wide association studies have led to the identification of novel mutations associated with LGS. The *TANC2* gene, encodes a synaptic scaffolding protein that interacts with other proteins at the postsynaptic density to regulate dendritic spines and excitatory synapse formation. The *TANC2* gene mutations were reported in neurodevelopmental disorders and epilepsy but not in LGS ever.

**Case presentation:**

Here we describe the case of a boy with LGS who presented with multiple seizure patterns, such as myoclonic, atonic, atypical absence, generalized tonic-clonic, focal seizures, and notable cognitive and motor regression. The seizures were refractory to many antiepileptic drugs. He got seizure-free with ketogenic diet combined with antiepileptic drugs. A de novo nonsense mutation c.4321C > T(p.Gln1441Ter) in *TANC2* gene was identified by the whole-exome sequencing and confirmed by Sanger sequencing.

**Conclusion:**

We described the first Chinese case with LGS associated to a de novo nonsense mutation c.4321C > T(p.Gln1441Ter) in *TANC2* gene, which would expand the clinical spectrum related to *TANC2* mutations and contribute to better understanding of genotype-phenotype relationship to guide precision medicine.

## Background

Lennox-Gastaut syndrome (LGS) is a severe epileptic encephalopathy in which the epileptiform abnormalities may contribute to progressive dysfunctions [1], characterized by polymorphic seizures and neuropsychological decline [2]. LGS may arise from multiple etiologies. In symptomatic cases, LGS most frequently occurs secondary to damage to the brain resulting from prenatal or perinatal insult, infection, malformations, or tumor [3]. However, approximately one fourth to one third of LGS cases have no clear etiology, among which some cases were found to be associated with genetic mutations thanks to increased application of next-generation sequencing in the field of clinical diagnosis.

A previous study systematically reported the genetic and clinical data for 20 patients with pathogenic *TANC2* mutations for the first time in 2019, as a follow-up of a recent meta-analysis of whole-exome sequencing (WES) data from 10,927 families with neurodevelopmental disorders (NDDs), which include autism spectrum disorder (ASD) and epilepsy [4]. The *TANC2* gene, located at chromosome 17q23.2-q23.3, encodes a synaptic scaffolding protein that interacts with other proteins at the postsynaptic density to regulate dendritic spines and excitatory synapse formation [5]. However, it is unknown whether *TANC2* mutations are associated with epileptic encephalopathy.

The present study describes a 1-year and 11-month old boy who suffered from LGS. By WES analysis, a de novo nonsense mutation was identified in *TANC2* and thus established the link between LGS and *TANC2* mutations.

## Case presentation

The proband was a 1-year and 11-month old boy at the time of admission. He is the third child with two healthy elder brothers of a non-consanguineous marriage, born after full-term by normal delivery, with a birth weight of 3000 g and allergy history of cephalosporin. No other members in the family have seizures. Nothing abnormal was observed in the perinatal period. Motor development and language development were mildly delayed before the onset of seizures, with an unbalanced gait and dual tone. The first two seizure episodes occurred within half a day when the temperature reached 39 °C at 1-year and 10-month old, which presented with generalized tonic-clonic, lasting for about 2 min per episode. Subsequently, the convulsion occurred twice during the following week without fever or other inducements. Twenty days later, the convulsion occurred again and presented as tonic of four limbs. Then the convulsion occurred more frequently (10–20/day) with varied epileptic seizure types such as myoclonic, atypical absence, atonic seizures a week before admission into our clinical center.

**Fig. 1:**
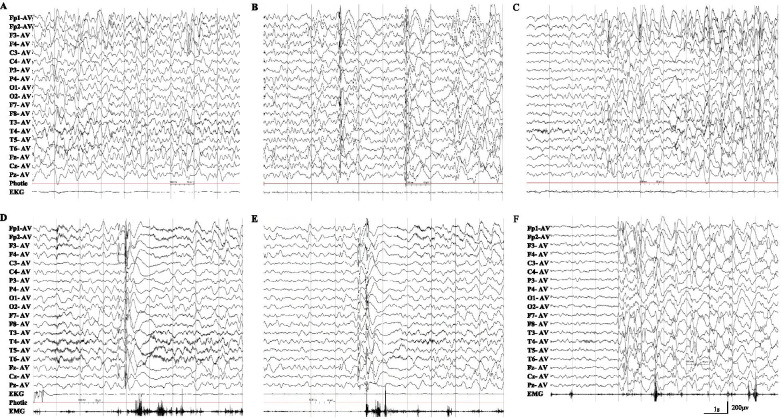
The EEG of the patient with *TANC2* mutation. **A** The background was slow, diffused θ and δ activities were seen; **B** Generalized slow spike-and-wave complexes during sleep; **C** Paroxysmal generalized 1.5-2 Hz spike-and-wave complexes with atypital absence seizure; **D** Generalized spike-and-wave complexes with atonic seizure and flattening in EMG; **E** Generalized spike-and-wave complexes with clonic seizure and EMG burst; **F** The re-checked EEG, generalized slow spike-and-wave complexes during sleep

His height, body weight, and head circumference were 85.0 cm, 11.8 kg, and 48.0 cm respectively at the admission. He had no obvious craniofacial dysmorphisms with fontanel closure. No other positive signs were found upon physical examination except slight gait instability. Results from routine examination including urine/blood/cerebrospinal fluid, blood/cerebrospinal fluid biochemistry, blood electrolytes examination, blood glucose, cerebrospinal fluid pathogens, metabolic screening and brain magnetic resonance imagery were normal. Video-EEG monitoring was performed for 3 h which showed slow background activity accompanied by a 2 ~ 3 Hz slow activity major in anterior area (Fig. 1A), interictal EEG showed a large number of generalized high amplitude 2 ~ 2.5 Hz slow spike-and–wave complexes (Fig. 1B). Two atypical absence seizures with generalized high amplitude 2.5 ~ 3 Hz spike-and–wave complexes lasting 7 s (Fig. 1C); two atonic seizures with 1-2 Hz polyspikes-and-wave complexes and electromyography flat (Fig. 1D); and several myoclonic seizures with 1-2 Hz polyspikes-and-wave complexes and electromyography burst were recorded (Fig. 1E). The myoclonic seizures disappeared and atypical absence and atonic seizures improved with combined treatment of sodium valproate and nitrazepam. Then focal and general tonic-clonic seizures were seen during sleep occasionally. Follow-up EEG showed a large number of generalized high amplitude 1.5 ~ 2.5 Hz slow spike-and–wave complexes in the slow background (Fig. 1F). Lamotrigine then was added as the third drug for combination therapy, and after which the focal and general tonic-clonic seizures were well controlled and atypical absence, atonic seizures reduced. However, subsequently epileptic spasms emerged more than 10 times a day especially when he was quiet or shortly after waking up. During the two-month follow-up, the child exhibited gait instability and cognition regression, the Gesell scale scored 55, including gross motor 54, fine motor 47, language 70, cognition 50, social development 54. Based on the aforementioned clinical manifestations, the patient was diagnosed with Lennox-Gastaut syndrome (LGS). He was seizure-free with ketogenic diet combined with antiepileptic drugs forementioned finally. The blood Ketogen level was maintained at about 3 mmol/L.

**Fig. 2:**
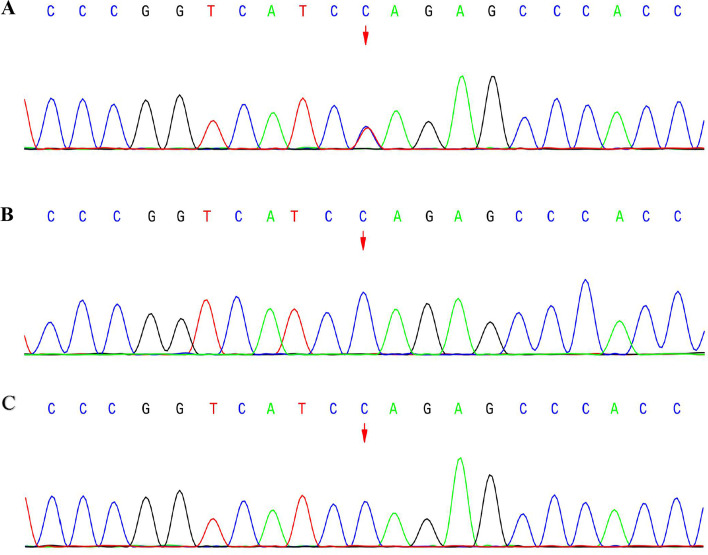
Sequence analysis of the *TANC2* gene in the family. The arrows show the mutation site, Heterozygous mutation of *TANC2* gene c.4321C > T (p.Gln1441Ter) was identified in the proband, none the parents carried the mutation. **A** The result of the proband; **B** The result of the father; **C** The result of the mother

To identify the potential pathogenic mutations, the proband and the parents’ genomic DNA was extracted from peripheral blood lymphocytes using the QIAamp® Blood Mini Kit (Qiagen, Hilden, Germany) and subjected to whole-exome sequencing (WES) on Illumina NovaSeq6000 (Illumina, San Diego, CA, USA). The read mapping, variant calling, genome annotation, and variant prioritization were performed as described previously [6], the variant was classified according to standards and guidelines of the American College of Medical Genetics and Genomics (ACMG) [7]. The results were also confirmed by Sanger sequencing. WES showed only one de novo heterozygous mutation c.4321C > T (p.Gln1441Ter) in *TANC2* gene (NM_025185.4) in the proband (Fig. 2). No other rare variants in any other genes associated with LGS reported previously were found in the patient. This nonsense mutation would lead to premature translation termination and has no allele frequency in several commonly used databases (i.e., dbSNP, gnomAD, ClinVar, HGMD). The in silico prediction score of GERP (Genomic Evolutionary Rate Profiling), LRT (Likelihood ratio test) and Mutation Taster is 5.29, 0 and 1 respectively which indicate pathogenicity. According to the ACMG clinical variant interpretation guidelines, the pathogenic evaluation of this variant was “PVS1 + PS2 + PM2”, which classified as “Pathogenic”. The genetic characteristics and pathogenetic analysis of the variant in *TANC2* were displayed in Table 1.

**Table 1:** The genetic characteristics and pathogenetic analysis of *TANC2* mutation identified in this study

Gene	Location	Nucleotide alteration	Mutation type	MAF	GERP	LRT	MT	ACMG values
*TANC2*	chr17:63420303	c.4321C>T	Nonsense	NA	5.29	0	1	PVS1+PS2+PM2

*MAF* Minor allele frequency, *GERP* Genomic Evolutionary Rate Profiling, *LRT* Likelihood ratio test, *MT* Mutation Taster, *NA* Not available, *ACMG* American College of Medical Genetics and Genomics

## Discussion and conclusions

Lennox-Gastaut Syndrome (LGS) is considered as a severe epileptic encephalopathy with onset in childhood, which implies that the epileptic activity contributes to mental problems and behavioral disorders [8]. It is defined by a triad of multiple drug-resistant seizure types, a specific interictal EEG pattern showing bursts of slow spike-wave complexes or generalized paroxysmal fast activity, and intellectual disability [8]. However, not all patients have all of the core seizure types (i.e., tonic, atonic, and atypical absences), especially at the onset [9]. The age of onset of LGS is typically between 1 and 7 years, most commonly between 3 and 5 years [10]. The long-term outcome for patients with LGS is generally poor and complete seizure freedom is unusual [8]. Its etiology is broken into six subgroups [11], among which genetic factors are getting more and more attention, where more than ten related pathogenic genes have been reported.

In the patient reported here, febrile seizures occurred twice at 1-year and 10-month old. Since then, he manifested frequent seizures and exhibited multiple seizure patterns including atypical absence, atonic, myoclonic, focal, general tonic-clonic and tonic. The video EEG revealed synchronous (generalized) discharge, with a large number of generalized high amplitude 2 ~ 2.5 Hz slow spike-and–wave complexes, spike-and–wave complexes, polyspikes-and-wave complexes in the background. The oral administration of sodium valproate, nitrazepam and lamotrigine made myclonic, focal and general tonic-clonic seizures free and reduced the frequency of atypical absence and atonic seizures, but epileptic spasms emerged frequently later, which indicated that it was a refractory epilepsy and had a partial response to sodium valproate combined with nitrazepam and lamotrigine treatment. The child developed mild delay in motor and speech before the onset of seizures. His cognitive and motor function regressed with the refractory epilepsy in 2 months follow-up. All these symptoms were consistent with LGS diagnosis.

There were no abnormalities in brain magnetic resonance imaging. Metabolic screening and cerebrospinal fluid etiology screening were both negative. A de novo c.4321C > T (p.Gln1441Ter) mutation in *TANC2* gene was identified in the child by whole-exome sequencing and confirmed by Sanger sequencing. The variant is located in a high conserved region and has a deleterious effect by in silico prediction. According to the ACMG clinical variant interpretation guidelines, the variant is classified as a pathogenic mutation. We considered the variant as the cause of the disease in this patient based on the genotype-phenotype association and its co-segregation with the disease.

### TANC2 function, clinical manifestations, and precision medicine

Our study and the review of the literature suggested an important role for *TANC2* in brain development*. TANC2* encodes a synaptic scaffolding protein that interacts with other proteins, especially PSD-95 (postsynaptic density-95), KIF1A (the primary motor protein for synaptic vesicles), and various subunits of the NMDA receptor (Grin1 and Grin2B), at the postsynaptic density to regulate dendritic spines and excitatory synapse formation [12, 13]. In addition, TANC2 deficiency leads to embryonic lethality, indicating the importance of TANC2 during embryonic development [12]. According to the transcriptomic profiles in the developing human cerebral cortex, *TANC2* is broadly expressed in many cell types but enriched in excitatory neurons and radial glia [4]. These functional investigations are consistent with our observation that truncating mutations in our report and others lead to malfunction in synapse-related neurological processes.

We observed severe epilepsy in our case, however, within a larger pool of 20 probands or affected siblings with *TANC2* disruptive variants, epilepsy was only observed for 11 individuals and the degree of epilepsy was variable [4]. Upon more careful evaluation of the individual mutations and their corresponding clinical manifestations, there was no apparent genotype-phenotype association, even though all the likely disruptive mutations lead to the missing of PDZ interacting motif, which is essential for its synaptic localization [4, 14]. The most consistent phenotypes associated with *TANC2* disruption, including intellectual disability (ID), speech-language delay, and childhood motor delay, are also observed in our case. Considering the still limited number of mutations reported so far, more reports in the future would be needed to expand the clinical spectrum related to *TANC2* mutations and to clarify the genotype-phenotype correlations (if any).

We also reported the treatment and the response of our patient in this case. Again only with increased number of future reports and thus accumulated database on genotype-phenotype relationships and corresponding drug response, can we start to realize the ultimate goal of precision medicine.

In conclusions, we performed a clinical and molecular genetic study related to a Chinese boy with Lennox-Gastaut Syndrome and found a potential causative mutation in *TANC2*. After reviewing the literature about the *TANC2* gene and function, we demonstrated that the gene plays an important role in neurodevelopmental disorder and epilepsy. To the best of our knowledge, this is the first report about *TANC2* mutation in LGS patients, which would increase clinicians’ understanding of the genetic profile of LGS and expand the still limited *TANC2* mutation spectrum as well.

## Data Availability

All data and materials mentioned in this manuscript, except patient’s private information, can be promptly available to readers by a reasonable request to the corresponding author.

## Abbreviations

ACMG: American College of Medical Genetics and Genomics
ASD: Autism spectrum disorder
EEG: Electroencephalogram
HGMD: The Human Gene Mutation Database
ID: Intellectual disability
LGS: Lennox-Gastaut syndrome
LRT: Likelihood ratio test
NDDs: Neurodevelopmental disorders
NMDA: N-methyl-D-aspartate
PSD: Postsynaptic density
WES: Whole-exome sequencing
